# Book Reviews

**Published:** 1830-09

**Authors:** 


### CRITICAL ANALYSES.

Quæ laudanda forent, et quæ culpanda, vicissim

Illa, prius, cretâ; mox hæc, carbone, *notamus*.—PERSIUS.

*An Inquiry concerning the Indications of Insanity, with Suggestions for the better Protection and Care of the Insane.* By JOHN CONOLLY, M.D. Professor of Medicine in the University of London.—8vo. pp. 496. John Taylor, London, 1830.

THE chief objects which Dr. CONOLLY has in view are to render the recognition of insanity less difficult, by showing in what it differs from those varieties of mind which approach the nearest to it; and to point out those circumstances which, even in persons decidedly insane, can alone justify various degrees of restraint. That the task which he has undertaken is one of no ordinary difficulty, will be at once acknowledged. Every attempt that has hitherto been made to draw a precise line between perfect sanity of mind, eccentricity of mind, and positive insanity, has failed, although the subject has frequently been considered by the first metaphysical and practical writers. That the same difficulty frequently exists in determining when personal restraint is necessary, and when it is not, has recently been proved by the contrariety of opinions which have arisen in particular cases that have attracted so much public and professional attention. It is much to be lamented that, where the medical student has so much to learn, he should, in this country at least, have so little opportunity of learning. When he enters upon the practical duties of his profession, no cases can fall into his hands that will cause him more perplexity, or that will so much endanger both his moral and medical reputation, as cases of insanity; and yet his previous studies must have generally left him quite unprepared for the difficulties with which he will here have to contend. We cannot, therefore, but agree with Dr. Conolly that the interests of the public greatly require that medical men, to whom alone the insane can ever properly be intrusted, should have opportunities of studying the forms of insanity, and of preparing themselves for its treatment, in the same manner in which they prepare themselves for the treatment of other disorders.

"It would be some compensation for the unavoidable evils of public lunatic asylums, if each establishment of that kind became a clinical school, in which, under certain restrictions, medical students might prepare themselves for their future duties to the insane. It is true that insane patients are not always in a state to be visited by pupils, and that a very strict discipline would be necessary to prevent disorder or impropriety: but such discipline is quite practicable; and such arrangements might be made as would at once guard those patients from disturbance whom disturbance might injure, and present a sufficient number of instructive examples to the student. In some cases, also, the change of persons and of conversation would be actually beneficial to the patients; and would only be what they are now accustomed to, during the visits of persons who come to see them from mere curiosity." (P. 7.)

We know how many plausible objections may be urged against any such system as is here recommended; but we think Dr. Conolly's suggestion might be acted upon without injury to the unfortunate patients, while the adoption of it must confer the most important benefits upon medical students, and indirectly upon the public, for whose service their professional information is acquired.

In his observations upon the present condition of lunatic houses and lunatics, Dr. C. assures us that he has "no wish to exaggerate the disadvantages of lunatic houses;" and yet we apprehend that he has dwelt upon these "disadvantages" with undue severity. The reprehensions that he has so freely bestowed may apply to a few, and we hope and believe only a few, of such establishments.

We must pass very cursorily over the several chapters which Dr. Conolly has dedicated to an analysis of the human understanding, and a description of its various degrees of perfection in different individuals: its inequalities, weaknesses, and peculiarities, which do not amount to insanity; and the modifications of intellectual activity and power by stimuli, disease, or age. The medical student, who is ambitious of acquiring practical information upon the important subject of insanity, must first make himself acquainted with the history of the human mind in general. He must know its healthy functions and natural modes of action: if he be ignorant of the numerous varieties of mental characteristics which belong to different individuals; if he have not a clear conception of the many deviations which exist from a healthy standard of mind, which still do not reach insanity, he will be as unequal to the task of judging of or treating mental diseases, as he would be of alleviating corporeal maladies without a due knowledge of the healthy structure of the body. We fully admit, therefore, the necessity for the discussion which Dr. Conolly has entered into, and we greatly admire the easy elegance of language in which he has conducted it; but we believe that many will be inclined to think with us, after they have perused the several chapters to which we have referred, that he has spread "a mite of *original* matter through a world of space."

Dr. Conolly believes that practitioners, "for the most part," neglect the important duty of considering, with all the caution which such a serious case requires, *whether or not the departure from sound mind be of a nature to justify the confinement of the individual, and the imposition of restraint upon him, as regards the use or disposal of his property.* What facts may have occurred to Dr. Conolly to justify so harsh and almost indiscrimate a censure upon his professional brethren, we know not. We think that the charge is incautiously made; but, in the present temper of the public mind, which has recently been most unjustifiably inflamed and misled upon the subject, such observations will doubtless be very popular. Dr. Conolly, however, we are certain, would spurn at any personal advantages which might arise from increasing the prejudices of the public against any members of his profession.

It is the opinion of Dr. Conolly, that, if we give a due consideration to the intellectual constituents of what is called sound mind, and take an enlarged view of its varieties, that we shall find, on coming to the examination of cases of undoubted insanity, that the departures from sound mind may generally, if not always, be recognised; and that the second and most essential part of the practitioner's duty, that of determining concerning the liberty or restraint of each individual affected with insanity, lies within narrower limits than he has been led to suppose.

"As a knowledge of the natural structure and functions of the body prepares him to detect impairment of either, so should he be prepared for the detection and management of diseases affecting the mental functions, by a previous acquaintance with the natural constitution of a healthy mind. Among the subjects comprehended in the studies of zoology and human physiology, this has seldom attracted a due, because a useful, share of attention; although the phenomena and the order of phenomena present no greater difficulties in the way of observation than those which he is accustomed to in his study of the functions of respiration, circulation, digestion, or reproduction; and are in no degree more hidden and mysterious even in their nature, though certainly not less so." (P. 36.)

Dr. C. first describes the healthy functions and natural modes of action of the human mind, and then traces mental impairment, from the slightest to the severest kinds. We agree with him that this is the mode in which the inquiry ought to be conducted. The general plan of investigation here adopted differs but little, indeed, from that followed by ARNOLD, CRICHTON, and other writers.

"By this consideration of the mental lesions, one after the other, and by contrasting each of them with sound or healthy mind, the conclusion has certainly to me appeared inevitable, that insanity never exists without such an impairment of one or more of the faculties of the understanding as induces, or is accompanied by, some loss of the power of comparing. The justness of this conclusion may now be tried by selecting different examples of insanity, in some of which one faculty, in some another, in some many, are affected; and by observing whether or not the affection of one or more faculties would alone account for the insanity, or whether such affection is only followed by insanity when the comparing power begins to be injured; and whether the nature and extent of the insanity depends, as I have stated it to be my belief that it does, on the nature or extent of that injury or impairment of comparison." (P. 306.)

Dr. Conolly does not dogmatically insist upon the infallibility of the definition of insanity which he has arrived at by the train of investigation he has adopted. He puts it interrogatively, whether we can approach any nearer to a definition of insanity than by saying, that *it is the impairment of any one or more of the faculties of the mind, accompanied with, or inducing, a defect in the comparing faculty?*

"In a former chapter, many examples were given of the sensations being diseased or false; but in which the patient knew them to be so, and retained his reason. Let us, in this place, attend to some of the cases in which the sensations are morbid, and the reason is not retained. In numerous instances the hallucination of the sense arises from an imagination previously over-excited; that over-excitement is disease, but not madness; it produces an hallucination, but, if the hallucination is known to be an hallucination, still there is no madness; if it is mistaken for reality, then the man is mad. An ambitious general, under Napoleon, believes that he hears the people salute him as their king. A Vendean commander sees Louis the Sixteenth in the balcony of the Tuilleries on a Sunday, and believes that the monarch calls to him by name, and creates him a marshal of France. An enthusiastic musician hears the singing of a choir and company of angels. In all these cases, such thoughts as perhaps not unfrequently have floated through the mind, are, from some disturbance in the functions of the brain, vividly represented, and too tenaciously retained. The ambitious general, in his reveries of possible greatness, has often, it may be, contemplated that, like his master, he might attain sovereign power: the Vendean commander, exaggerating his own merits, has long thought himself deserving of high distinction: the composer is familiar with harmonious mental conceptions: but something has happened to give to these imaginations unusual strength. In each case, then, there is a diseased state of the mind. But it is the insane belief which causes these men to be considered mad. From whence does that insane belief arise? Doubtless, from a want of the power to compare things which are, with things which are not. Whether this want of power arises from the force of the morbid impression, or from the want of attention to present circumstances, or from the defective manner in which past things are recalled, still it is the want of that power which leads to the madness. The general hears the voices of the people; if he looked out of the window he would see that no assembled crowd was there to hail him king: but he cannot see this, for his attention is not in his power; he can only hear the solicitations of the people; he cannot, therefore, compare the deception of some sense with the evidence of another sense; and he becomes inevitably mad. The Vendean commander sees the king in the balcony, and all the people assembled on the same occasion see the king also; but the Vendean hears the king call to him; which the rest of the people do not hear: it is a morbid or false impression; if he looked around him, he would find that no impression of the king's words had reached any ear but his; but this he cannot do, and he returns to his house full of his new dignity. He does not remark the absence of any further notice of him; the silence of the newspapers; the neglect of the court. He cannot, perhaps, remark these things, he cannot attend to them, and therefore he has nothing which he can compare with his hallucination: he makes no comparison, and he becomes a madman on the subject of the hallucination." (P. 307.)

The senses are subject to various delusions, particularly the senses of sight and hearing; sometimes the sense of taste; more rarely the sense of touch. A thousand instances are related of persons who have imagined some change to have been wrought in their appearance or structure. Men have believed themselves to be converted into teapots; into barrels which were rolled along the street; or into a town pump, to which no rest was given from morning until night. A respectable tradesman fancied himself metamorphosed into a seven-shilling piece, and went round to his customers to entreat that, if his wife should present him in payment, they would not give change for him. Others have imagined some animal was in their stomach, or that many armed knights were battling there, and some have thought themselves possessed of a double set of bowels, productive of discordant and highly inconvenient movements.

"In all these cases, for they all admit of one explanation, there is first a morbid sensation. We have seen that a morbid sensation does not constitute madness. But this impairment of sensation becomes, in certain cases, productive of, or accompanied by, a loss of the comparing power; either productive of the loss by its force, or accompanied by it in consequence of some further disease, as of the attention or memory, and then there is madness. When a man can see, hear, eat, drink, and sleep, and yet affirms that he is a teapot, it is plain that he either has no sensation but that which gives him the impression of being a teapot, or that he cannot attend to other impressions so as to compare them with that morbid impression. If comparison were made by him, his belief would be at an end; for a teapot neither eats, nor drinks, nor sleeps. A man who, sitting quietly in his room, believes himself to be a barrel rolled along the street, is, in the first place, the victim of a morbid sensation; but if he could compare the sensations arising from the objects actually around him, with his morbid sensations, he would detect the delusion, and laugh at his morbid feelings. He cannot do this, either because he feels nothing but his morbid sensations, or cannot let his attention rest on other sensations; and, in either case, he is mad, because he cannot compare one sensation with another." (P. 310.)

In the disturbance of a fever, the image of a friend is seen sitting by the patient's bed, and the soothing tones of his voice are heard; but the patient stretches out his hand, and finds that no hand meets his, and that the chair is empty. "The evidence of the sense of touch is compared with the evidence of the senses of sight and hearing, and the delusion is at an end. Cases have been observed in which, the delusion not imposing on the mind, patients have been amused to find the solitude of their sick chamber relieved, at the time of every evening exacerbation, by a party of friends, the presence of whom was known to be a mere hallucination. It is when the delusion is stronger, or the comparison of other circumstances with it imperfect, that madness begins." M. Falret, in his work on Suicide, mentions the case of a lady who, when she merely looked at her skin, often thought it scaly, like the skin of a fish, but used immediately to rectify this false sensation by the sense of touch, which was unimpaired. Here was a morbid sensation, rectified by the act of comparison. If the power of comparing had not remained with her, she would have been mad with respect to the state of her skin.

"The celebrated Pascal was the subject of a false sensation, representing to him the edge of an immediate and fearful precipice. To allay his apprehension of falling down it, his attendants were accustomed to place a chair near him, in the direction or situation of the supposed precipice; and he then compared what was done with what appeared to him, and drew the just conclusion that a chair could not stand upon air, beyond the brink of a precipice, and that he was therefore not in real danger. As long as he was able to do this, for he could not always do it, he was enabled to get the better of the hallucination; whenever the comparison could not be made, the delusion yet remaining, he was not sane on the subject of the precipice." (P. 315.)

Dr. Conolly adduces various other instances, and introduces many arguments, for the purpose of showing that the definition he offers will bear a strict examination. We have no doubt that it will apply as generally, perhaps more so, than any other that has been proposed. We believe, however, with Dr. Haslam, that it must be impossible to discover an infallible definition of madness, as it is an attempt to comprise, in a few words, the wide range and mutable character of this Proteus disorder.

We now pass to that part of Dr. Conolly's work to which the practitioner will refer with the most anxiety, the application of the inquiry to the duties of medical men, when consulted concerning the state of a patient's mind. When a practitioner is consulted concerning a patient supposed to be labouring under mental derangement, he cannot avoid deriving first impressions from the representations of those who apply to him. He must guard himself, however, as much as possible against suggestions which are designed to influence his proceedings. He should *never* fail to pay an early visit to a patient suspected to be insane.

"The whole duty of a medical man in such cases may be resolved into two parts:

"1. To determine whether the individual in question be of sound mind.

"2. To give an opinion concerning the treatment required, and especially concerning the necessity of restraint, and the degree and nature of the restraint." (P. 365.)

If the patient is disturbed by any cause of agitation which we can detect, we should refuse to give a final opinion concerning the state of his mind: we should even be careful that he has undergone no recent disturbance; and should present ourselves to him with the same openness of manner with which we approach the bedside of a patient in fever. The good effects of this mode of accosting those who have been treated with harshness, Dr. Conolly has often witnessed.

"The fact of the patient's madness can only be established by certain tests of the manner in which his intellectual faculties are exercised; and these tests are to be found in his appearance, in his dress, in the known physical accompaniments of madness, and in his words and actions. *That* is the medical question. The *next* is a medico-legal question, and turns wholly on the disposition of the patient to injure himself or his property, or to injure others and their property; and on the probability of such a disposition, though not manifested, being suddenly developed. On the first question hangs the medical treatment and superintendence; on the second, restraint, confinement, deprivation of authority, and control over property. Medical care and superintendence may be necessary in every case; but the mistake has been to conclude, that restraint and the other circumstances are also necessary, which they certainly are not.

"The strong association formed between certain judgments, and certain associated ideas, and certain external acts, including those muscular actions which give expression to thought independently of speech, often portrays the madness, and its character, plainly enough, in the features and movements of the patient. When a man, who has not recently been exposed to great cause of irritation, is found indulging in great violence of gesticulation and passionate exclamations, or when he sits motionless, and does not regard those who come into the room, and does not reply when spoken to, there can seldom be any doubt that such a man is not in his right mind. If, approach him in what manner he may, the practitioner finds that he cannot be tranquillized, or that he cannot be roused, of that man's insanity there can be no doubt or question: but the practitioner must first vary his modes of approach; must do all that he can devise to allay what may be temporary, though inordinate passion, or to soothe what may be deep and speechless, though temporary grief, before he comes to this conclusion." (P. 373.)

That insane persons are often very dexterous in concealing, for a time, any indications of their lamentable malady, is notorious; but, whatever deception we may suspect, if the patient complains in quiet terms, asserts his sanity without vehemence, assigns interested motives for the conduct of his relatives without passion, or with but natural warmth, we must not allow ourselves to be so influenced by a good opinion of any other party as to be heedless of this demeanour. The case should engage our most serious attention.

"The practitioner should be aware that there are many individuals whose ordinary manner is weak and foolish, but who are yet quite equal to the management even of important concerns; and that there are others whom the least hurry discomposes. No one has authority to insist on immediate decision, when the decision is in any degree doubtful; and if the practitioner pronounces a decision before he has really satisfied his mind, he has no right, from that time forward, to look upon himself as an honest man." (P. 389.)

The result from careful inquiries must govern the whole treatment of the case; both the administration of medicine, as well as the application of restraint, against the abuses of which Dr. C. particularly directs his remarks.

"The medical treatment must depend on the bodily state of the patient; for violence, and incoherence, and raving, may all arise, in different instances, from states of the body entirely opposite to one another, and requiring an opposite treatment. The restraint necessary is determined by the violence or folly which is present, or by the violence or folly known to be meditated." (P. 389.)

In various instances restraint may be necessary, but it is too much forgotten that it might often be effected without the confinement of the individual among mad people. Restraint, however, either of the person or the management of his own affairs, can never be justifiable except by probable danger to the person of the patient, or to others, or to his property, or to the property of others. But it may often be proper to *watch* a patient very narrowly, which may be effected without restraint; the latter being added when watching proves it to be necessary. Men may entertain various delusions which, being neither dangerous to themselves or others, do not require that they should be restrained by confinement.

"There are cases in which the madness is of a nature to threaten others, or the individual himself, with bodily danger; the thoughts of the patient turning much on scenes of destruction, and cruelty, and blood. The looks, or the language, or some outrage already committed, commonly reveal this character of the malady in cases in which it prevails; and, when once it is revealed, every thing that security demands must be at once resorted to, and persevered in until all danger is past. To say when the danger is past, is one of the most difficult things among the responsibilities of practising among the insane." (P. 444.)

Practitioners are frequently called upon to determine concerning the actual state of mind of an individual, who having committed some great crime, the question is whether he should be made responsible for it with his life.

"No question can well be more serious; and it is not only embarrassed by all the common difficulties of ascertaining the exact state of the mind, but by the additional difficulty of determining whether or not, whatever may be the present state of the prisoner, he was mad at the time of committing the crime; a thing, of course, hardly admitting of a satisfactory decision. On looking over the remarkable trials in which crimes have been perpetrated under these circumstances, it is impossible not to see that men have been executed for them, whose eccentricities have been greater than those which, in other cases, have been looked upon as justifying restraint of person and deprivation of property. The determination of the existence of the insanity is the medical question, and it must be determined in the usual manner; sufficient time and opportunities being demanded for making such an investigation as the serious nature of the case requires. The criminal may be mad on one subject only, and that may be the subject of his crime; he may be mad at intervals, and might be mad when he committed the crime; but I believe the legislator will sometimes be inclined to consider punishment justifiable in such cases, for the protection of society from other monomaniacs, or from those occasionally insane, whom yet such an example may restrain. At all events, a man who has once in his madness committed murder, or who has attempted to commit murder, must be secured for the remainder of his life against a repetition of such an act; and the more doubtful, transitory, or limited his insanity, the more necessary will it be to insist on that confinement which all would agree was required in plainer cases. The prevention of the crime is what might be more attended to than it is; for the act is often that of a mischievous idiot, whose restraint would appear necessary long before he proceeded to such daring or cruel mischief, if some erroneous notion respecting his not being mad, or his being considered idiotic, did not blind medical observers to the consideration of the dangerous character of his imbecility." P. 450.)

In the last chapter, Dr. Conolly offers various "suggestions for the protection and care of the insane;" for which we must refer to the work. Amongst other regulations, it is recommended that all persons confined or in any way restrained on account of insanity, should be allowed the use of pen, ink, and paper, and that they should be permitted to address sealed letters to the governor of the asylum, or to some person in authority over them. We believe that such an indulgence would very rarely be advantageous to insane persons, and we are certain that it would frequently be decidedly mischievous. A passion for scribbling is common among this class of patients, and, in several instances which have fallen under our own observation, the mind of lunatics has been kept in a high state of excitement, so long as they were in possession of materials for writing. Such an indulgence would only enable them to annoy others, and would lead them to torture their own minds to describe imaginary complaints and grievances, whilst it would, at the same time, keep their imaginations eternally on the rack upon the subject of their hallucination, and thus strengthen and confirm their malady.

Dr. Conolly's work proves him to be a man of talent and liberal education: his style of composition is fluent and elegant. One fault, however, pervades it, which cannot escape remark; a want of conciseness. In various parts of the work the argument is enervated by prolixity of language. We are somewhat doubtful of the utility of the work as an instructive volume to the medical student, the great point to which every writer should aspire. We fear that, when the practical inquirer, who has consulted previous authors on the subject of insanity, rises from the perusal of these "Indications," and reflects upon what he has read, he will not find that much additional information is impressed upon his mind which can serve him in the actual performance of his professional duties.

---

*The Influence of Climate in the Prevention and Cure of Chronic Diseases, more particularly of the Chest and Digestive Organs: comprising an Account of the principal places resorted to by Invalids in England, the South of Europe, &c.; a comparative estimate of their respective merits in particular Diseases; and General Directions for Invalids while travelling and residing abroad. With an Appendix, containing a series of Tables on Climate.* By JAMES CLARK, M.D. Member of the Royal College of Physicians in London, &c. Second Edition, enlarged. 8vo. pp. 400.—Underwood, 1830.

BOTH by English and foreign journalists the first edition of Dr. CLARK's work was hailed as a very valuable addition to the medical literature of the day. The rapidity of its sale is a proof that the general opinion of its merit is equally favorable. It is one of the very few professional works which embodies much instructive information that must be peculiarly acceptable to the medical practitioner and student, and at the same time be highly interesting to the general reader. During the short period that has elapsed since the publication of the first edition, Dr. Clark has availed himself of every additional information he could procure, relating to the part on Climate. He has again carefully examined the principal places in England frequented by invalids, and from the observations he has made, and the information he received on the spot from authentic sources, he has enlarged and materially improved the account he formerly gave of these places.

To the only article on the climate of the northern Atlantic, in the former edition, (that on Madeira,) he has, in the present, added a few observations on the principal islands in that ocean, occasionally resorted to by invalids from Europe, viz. the CANARIES, the AZORES, the BAHAMAS, and the BERMUDAS. A pretty full account of the West

Indies is also now given, of the true character of the climate of which the author has reason to know much misapprehension has existed, from which great practical evil has resulted. Of some places in the south of Europe Dr. Clark candidly confesses he is not yet able to speak with confidence.* Lisbon, however, he considers an improper residence for consumptive invalids. From numerous medical friends residing in many of the places noticed, Dr. C. has received strong testimonials of the accuracy of the account he has given of their respective climates, and of their influence on diseases; and in letters which he has received from Madeira and Nice, it is stated that the cases sent to these climates during the last season have been better selected than on any former occasion. No man can have stronger inducements, therefore, to continue an investigation commenced under such favorable auspices. Much public benefit has been already conferred, and it has been liberally acknowledged.

The article on England has been entirely re-written; but as the additions it contains are necessarily incorporated with the description of it, of which we formerly gave so copious an analysis,† and as they would scarcely admit of separation, we shall pass on to the consideration of the Atlantic Islands. The climate of the North American continent differs materially in its physical characters from that of Europe and Africa. The summer heat is much greater, and the winter cold much more intense, under the same parallels of latitude, on the American shores than on those of Europe. The western Atlantic is also more stormy than the eastern. It appears from comparative meteorological observations, that while the winter is here nearly 6°, and the spring 2°, *colder*, the summer is 3° *warmer* than at Barbadoes. The autumn temperature is the same at both places.

"The explanation of the high temperature of the Bahamas, during the two latter seasons, is probably to be found in the frequent occurrence of southerly winds during that period of the year, and in the less degree of regularity of the trade winds at these islands than within the tropics. In the winter and spring, however, the temperature is considerably lower, and this is the period of the year which chiefly interests us in our present inquiry.

"The Bahama islands, generally speaking, are not unhealthy;

* Dr. Clark expresses his regret that the work of the late Dr. Hennen, "Sketches of the Medical Topography of the Mediterranean, &c." was not sooner published, as the observations of that accomplished physician would have been of great assistance to him in this part of his work. It will now, however, supply any deficiency.

† London Med. and Phys. Journal, October 1829, p. 332.

although there is a considerable difference in this respect, between the different islands. That of New Providence, in which is the capital, Nassau, the only town in the colony, is not by any means one of the healthiest; on account chiefly of some swampy ground which it contains. The small island, called Harbour Island, close to Eleuthera, one of the largest of the group, is esteemed particularly healthy, and forms the chief resort of invalids and convalescents from New Providence. There are several other healthy spots, as on the island of Abaco; but at all these places there is a great deficiency of accommodations, and, moreover, they are sixty miles distant from Nassau, the only place where medical advice is to be found.

"The most prevalent diseases, are fevers, chiefly of the intermittent and remittent character, and bowel complaints; cholera is not uncommon, and occasionally the Bahamas are visited by epidemics of the yellow fever; but the two first-mentioned diseases are by far the most prevalent.

"From the above description, it appears evident, that the Bahama islands are not well calculated for the generality of invalids. I am disposed to think the climate is not suited for consumptive patients, on account of the rapid changes of temperature, and the prevalence of winds often of a dry, cold, character. At the same time, persons having connexions in these islands, who could render their residence more advantageous, by affording the means of choosing a favorable station, and thus diminishing the inconveniences of the climate, might pass the winter there safely; and residents in the West Indies might derive considerable benefit by a change to these islands for a few months during this season. The wet and dry seasons occur pretty regularly at the same periods of the year, as within the tropics, and the same rules which are laid down, respecting the arrival and departure of invalids to and from the West Indies, and their conduct while there, are generally applicable to those visiting the Bahamas."

Bermuda, upon the whole, Dr. C. considers to be a healthy place. It is not subject to any endemic disease. During the autumn, occasional fevers, of a character resembling those which form the scourge of the West Indies, prevail with great violence. This, however, is by no means an annual occurrence. Bowel complaints are the most common diseases. Consumption is frequent. The cool season, from October till May, is the most healthy, and the only part of the year during which this climate is at all suited to invalids. One of the principal objections to Bermuda, as a winter residence for pulmonary invalids, is the prevalence of strong winds.

"Provided, however, that domestic circumstances rendered Bermuda a convenient residence, even consumptive invalids, with a little care to avoid exposure to the irritating north-west winds, might pass the winter safely, and, perhaps, with benefit, in this climate. By consumptive patients, I mean those predisposed to consumption. When this disease is established, it is found to run its course more rapidly here than in England. There are many beautiful spots in these islands, where, protected from the northerly gales by the cedar-clothed hills, the invalid might find sufficient space to enjoy exercise in the open air, almost every day during the winter. The neighbourhood of the little town of Hamilton, situated nearly in the centre of the islands, affords the most favorable situations for such a residence."

Dr. Clark has not been able to collect sufficient information to enable him to speak with confidence of the physical qualities of the CANARIES and AZORES, or of its influence on disease.

The WEST INDIES. These islands are occasionally recommended as a winter residence for pulmonic patients, and some other chronic diseases; but neither the physical characters of their climate, nor its influence on disease, seem to Dr. C. to be generally known or understood by medical men in this country. He confines his observations to the Caribbean islands which are those best suited to invalids, and most likely to be frequented by them. Of these islands, generally speaking, the high are the more healthy, the low and marshy situations very unhealthy, Barbadoes, St. Vincent, Antigua, and St. Kitts, are preferable to all the others for invalids.

"The mean annual temperature of the West India Islands, near the sea, is about 79° or 80°. The mean daily range is only about 6°; and the extreme annual range is not more than 20°. The mean temperature of the sea, at considerable depths in the vicinity of these islands, is 80°; and this is also the temperature of the springs near the level of the sea in Jamaica, as noticed by Dr. Hunter. The mean temperature of some of the habitable spots of the mountain ranges is probably not more than 65° or 70°. The mean temperature of the seasons, according to the European division adopted in this work, is at Barbadoes as follows: Winter, 76°7; Spring, 79°; Summer, 81°; Autumn, 80°."

The winter and early part of the spring is in general remarkably dry, and the weather fine. The summer is dry and hot, and autumn the season of the heavy rains. In the lower part of the Caribbean islands very little dew is deposited. From the account given of the climate of the West Indies, it appears evident that it is an improper one, generally speaking, for consumptive patients."

"It is too hot during the night; and, during the day, the high temperature and cloudless skies almost entirely defeat one of the chief objects for the attainment of which the invalid migrates to a warmer climate; I mean exercise in the open air. He could scarcely venture to take exercise, even on horseback, after seven o'clock in the morning, during the coolest season; and, as there is hardly any twilight within the tropics, he would not be able to enjoy the coolness of the evening, in this way. If we have found cause to condemn Italy as a summer residence for consumptive patients, there seems no just reason why we should commend the West Indies, even in winter, the temperature of which is above the summer temperature of any place in the south of Europe. If to this consideration we add the numerous privations, annoyances, and discomforts which are almost inseparable from a residence in the West Indies, I think we might almost be justified in erasing these islands from the list of places suited to the phthisical invalid. Among other contingent disadvantages may be mentioned the difficulty of procuring houses in proper situations, the expenses of living, the annoyance of musquitoes, sandflies, &c.

"If to these objections, founded on an impartial consideration of the nature of the climate and of the disease, we add those of a more conclusive nature, derived from the experience of medical men, I conceive the question of the propriety of sending patients labouring under confirmed consumption to the West Indies, will be set at rest for ever.

"In the first place, I may remark, that tubercular phthisis is by no means rare, even among the white inhabitants of the West India islands, while it is of frequent occurrence among the black; and it is not uncommon for individuals affected with this disease to migrate in search of health to a more northern climate.

"These circumstances, however, although properly noticed here, are not adduced as arguments against the propriety of sending consumptive patients to the West Indies; because we find phthisis prevailing, in a greater or less degree, among the natives of every civilized country in the world. But a very different conclusion must be drawn from the fact confirmed to me by numerous medical friends, who have resided in the West Indies—that consumptive cases sent thither proceed much more rapidly to a fatal termination than in temperate climates. And indeed, this is what we should expect, *a priori*, from considering the nature of the disease, and the well known influence of the summer climate of the south of Europe on its progress. I am not, however, prepared to maintain that cases of consumption do not occasionally present themselves, in which, even in the advanced stages, a temporary residence in the West Indies might not prove useful. But I do venture to affirm, that such instances are of comparatively rare occurrence, and I would scarcely attempt to designate them. If there are any such, they will be found among the more chronic examples of the disease, which occur about the middle period of life, and which are attended with little constitutional excitement."

The opinions of various writers are referred to, to shew the injurious effects of the West India climate on confirmed consumption. Those who have had the best opportunities of judging, are still somewhat undecided with respect to the influence of this climate on persons predisposed to, or threatened with, consumption. The affections of the chest most likely to derive benefit from a residence in the West Indies, are chronic diseases of the bronchial membrane, occurring in persons otherwise of a tolerably sound constitution. In stomach complaints, the West Indies is in general unfavorable. Chronic rheumatism has been ranked among the diseases that are benefited by the West India climate; but Dr. C. suspects more from theoretical reasoning than practical experience. In scrofula, affecting the external parts of the body, the climate generally proves serviceable. Dr. Ferguson speaks in strong terms of the good effects which he has observed this climate to produce in scrofulous diseases. With children and young growing persons the West Indies does not agree.

"There are also certain constitutions to which the West Indies will prove injurious, whatever may be their disease. Of this class are persons of weak irritable constitutions, or with irritable bowels, or deranged digestive organs generally, or with an irritable skin, or subject to cutaneous eruptions of an irritable character, or to copious perspirations; persons who suffer from severe headachs, or have any hereditary disposition to cerebral disease, or to insanity; and plethoric fair people generally."

The four islands above mentioned afford all the advantages of the country and climate, and fewer of the disadvantages, than most of the other islands. In many respects Barbadoes is superior to all the others, but St. Kitts, one of the most beautiful of the West India islands, possesses its advantages in some instances. A few interesting remarks are made relative to the management of the invalid during his voyage to, and residence in, these islands. Upon these subjects, very contradictory, and often very erroneous, counsel is given, and therefore Dr. C.'s directions are the more necessary and valuable.

---

*On the Diseases and Injuries of Arteries, with the Operations required for their Cure; being the Substance of the Lectures delivered in the Theatre of the Royal College of Surgeons in the Spring of* 1829. By G. J. Guthrie, F.R.S., Professor of Anatomy and Surgery to the Royal College of Surgeons, Surgeon to the Westminster Hospital, to the Royal Westminster Ophthalmic Hospital, &c.—8vo. pp. 416. Burgess and Hill, London, 1830.

(Concluded from p. 65 of our Number for July.)

The *effects arising from the application of a ligature to an artery* are next considered. Mr. Guthrie's observations have not led him to believe that a coagulum is absolutely necessary to the permanent closure of the artery, although it certainly assists in maintaining it.

"The artery is supposed to contract gradually up to its first collateral branch, but this depends entirely on the use for which the branch is required. In cases of amputation at the middle of the arm, the artery will go on diminishing in size up to the subscapular branch; the circumflex arteries diminishing in proportion, in consequence of their being so much less necessary than before the operation. The rule, as a general one, will however hold good in an operation for aneurism, but I do not believe it to be so important as has been supposed. I have seen several instances in which the artery has remained pervious below the collateral branch, the next immediately above the part where the ligature has been applied. Neither will the presence of a collateral branch immediately above where the ligature has been placed upon the artery always interfere with the consolidation of the wound, and the closure of the canal of the vessel. It may impede the process, and render it for a time less safe, and in some instances it may prevent it altogether. I have so often seen large arteries heal after division close up to the giving off of a considerable branch, that I can consider them to be always capable of doing so, provided they are naturally sound. If they are not sound, it is very doubtful what process may take place; but it will be less likely to be a healthy one, if interfered with by the greater proximity of the circulation. The power which suppresses hemorrhage in a bleeding artery, resides in the very extremity of the vessel itself." (P. 161.)

The ligature should be round and small; and the smaller the better, provided it be strong enough. The surgeon should bear in mind that there is no security for the patient until after the ligature has come away, unless it is retained an inordinate length of time, from having included some substances which do not readily yield under irritation; such as the extremity of a nerve, or a slip of ligament, which is not compressed in the noose of the ligature.

"The advantages to be derived from the application of a small ligature, from the least possible disturbance of the surrounding parts, and from absolute quietude, whilst the healing processes are going on, must be so obvious as to require no further observation. Several cases will be adduced hereafter, in which secondary hemorrhage was the consequence of motion of the limb at too early a period; and an undue interference with the ligature, by pulling at it, cannot be too earnestly deprecated, as giving rise to it in a similar manner.

"When secondary hemorrhage occurs to any extent, either through ulceration of the artery, or from the extension of ulceration or sloughing of surrounding parts to it, a ligature must be placed upon it nearer to the heart, and as far above the mischief which has taken place as the collateral circulation will permit; but the observations on the appearance of the blood and the manner in which it flows, (made at page 137,) should be strictly attended to. Many secondary hemorrhages may be restrained, and ultimately suppressed by moderate pressure, and the ligature should only be had recourse to, when the application of pressure would appear to be, or has been proved to be, insufficient for the purpose." (P. 166.)

*On the Application of a Ligature on the Artery below the Aneurism.* Mr. Guthrie is disposed to concur with Bichat in attributing the origin of this operation to Desault, who would appear to have proposed it to his class about sixty years ago. Boyer, in the account which he has given of it, says that the first attempt to cure an aneurism on this principle was made by Vernet, who tried it by compression on the upper part of the femoral artery, but below the tumor, which, however, increased so rapidly in size, its pulsations also becoming stronger, that he was obliged to give it up, and confine himself to palliative means. Deschamps performed the first operation, but without success, in a case of femoral aneurism, in a man sixty years of age. The second operation on this principle was performed by Sir Astley Cooper, in a case of aneurism of the external iliac artery, which extended into the abdomen, as high as the internal iliac. The femoral artery was tied between the origins of the epigastric artery and of the profunda. The pulsation continued, but the tumor did not increase in size after the operation. The ligatures separated favorably. The aneurism diminished so considerably, that it was conceived, if its diminution continued, it would in a short time be possible to tie the external iliac above the tumor. The patient went into the country to recruit his general health, when the aneurism burst into the inguinal canal, and he died in consequence of the extravasation of blood into the cellular membrane of the pelvis and the scrotum. In this instance the femoral artery was tied below the origin of the epigastric and circumflexa ilii arteries: a current, therefore, Mr. Hodgson says, continued to pass through the sac into these vessels: consequently the blood was not at rest in the aneurism, and did not coagulate. After the ligature of the artery, the blood was transmitted more readily through the internal iliac than through the arteries which originated below the aneurism, namely, the epigastric and circumflexa ilii. "The contraction of the sac, therefore, appears to have been the consequence of the diminution of the stream which passed through it, in the same manner as an aneurism contracts, although a current enters it after a ligature of the artery at a distance from the disease." The parts were not examined after death.

The third operation on the femoral artery below the tumor was performed by Mr. White. No diminution of size was perceptible on the application of the ligature, and it was unsuccessful in consequence of the inflammation and sloughing which followed. This operation was performed in consequence of the success which was *stated* to have followed its revival by Mr. Wardrop in several cases. Of these cases Mr. Guthrie gives an abstract, and he also alludes to the theory and opinions published by Mr. Wardrop in 1828. Mr. G. very properly states that, since the publication of Mr. W.'s book, circumstances have taken place which have made a great alteration in the success supposed to have followed several of these operations.* Mr. Wardrop, in his observations on these cases, seems to consider that the cure is accomplished on the same principles as in the Hunterian operation for aneurism, viz. by the stagnation and subsequent coagulation of the blood in the sac, aided by the contractile power of the artery and sac. Mr. Guthrie shows, however, "that the theory on which these operations are founded is not the same, and they cannot therefore, with propriety, be compared." But, in impugning the theory on which this operation is founded, Mr. G. by no means intends to deny its utility, or the necessity for its performance. It ought, he says, to be done in every case of carotid aneurism, in which there is not space for the operation nearer the heart.

Mr. Wardrop, relying on the correctness of the theory by which it is supposed that the cure of an aneurism took place when the artery was tied beyond the tumor, has proposed to extend it to aneurisms below which a ligature cannot be applied, such, for instance, as aneurisms of the arteria innominata; and on the principle that, if the passage of a portion of the blood which usually passed through an aneurismal tumor was cut off, by establishing a barrier beyond or below it, by ligature, the remaining portion would coagulate.

"He proposes in a case of this kind, therefore, to place a ligature first on the carotid, and then, if necessary, on the subclavian artery. I have already endeavoured to prove that a barrier

* Mr. Lawrence, in his surgical lecture (Med. Gazette, May 15th, 1830,) speaks very guardedly upon this subject. We require, says Mr. L., a considerable number of facts more than we at present possess, in order to determine the question whether tying the arterial trunk beyond the aneurismal tumor be capable of leading to its cure.—Ed.

formed beyond the tumor will not have the effect which he has expected from it, unless it is accompanied by an inflammatory action which is communicated to the sac, and through which the coagulation takes place. The preparations I have referred to, and the various cases I have instanced or alluded to, seem to put the point beyond doubt. Indeed, if it were not sufficiently established, Mr. Wardrop's own book furnishes further proof, which cannot be disputed.

"In the case of Mrs. Denmark which he published, supposing it to be a cure of aneurism of the innominata, which had been effected by a ligature placed on the subclavian artery, but who has since died of the disease, the fallacy of his theory seems to me to be most obvious." (P. 186.)

Mr. Guthrie concludes the subject of the application of a ligature below the aneurism by the following summary.

"1. Whenever the operation for aneurism succeeds from placing a ligature below or beyond the tumor, it does so by giving rise to inflammation in the aneurismal sac and in the artery both above and below it; and unless it does this it fails.

"2. That this operation, as well as all others, is exceedingly dangerous in the vicinity of the heart, from the facility with which the inflammation may be communicated to that, as well as to the neighbouring organs.

"3. That it will not effect a cure, in cases of aneurism of the innominata or arch of the aorta, although it may give temporary relief by the partial diminution of the tumor.

"4. That, being as likely to destroy the patient as to give this relief, it ought never to be performed until the life of the patient is in extreme danger from the size of the tumor, when the person may have the opportunity of choosing between a more sudden death or a temporary relief; but the chance of a cure should never be calculated upon.

"I have watched two cases of aneurism, supposed to be of the innominata, for the last two years, on both of which it had been proposed to perform this operation, but which the patients refused to undergo. They are still nearly in the same state; and although in many instances the disease proceeds with rapidity, it is in others slow in its progress, occasionally receding and again increasing in size, until at last a new impulse seems to be given to it, which tends rapidly to a fatal termination. It is then only that an operation of this nature should be thought of, and the result, even as to temporary relief, must always be very doubtful." (P. 208.)

*Of Wounds and Injuries of Arteries.* A variety of experiments have been made on the arteries of dogs and horses, to observe the manner in which the processes take place in them to effect a cure, or which precede the dissolution of the animals. This has been done to elucidate the manner in which these processes take place in man under similar circumstances. The analogy is not satisfactory, as the injuries of arteries in man do not follow the same course as in animals. The processes of reparation in man are by no means so favorable, as far at least as observations made on injuries of arteries will enable us to judge.

"When an artery is cut transversely in man, to one third or fourth of its circumference, it forms the same circular opening as in animals; and, if the artery is large, the bleeding usually continues until the person faints, or it is arrested by pressure. The difference, however, between the arteries of man and of animals is here most strongly exemplified. In an animal, the bleeding will probably be restrained altogether, without any assistance from art. In man, even with the best assistance in the way of compression, unless the circulation be altogether stopped, hemorrhage will in all probability recur, and, if the external opening be closed, a spurious aneurism will be the consequence." (P. 213.)

In man, no reliance can be placed on the efforts of nature in healing a wounded artery. It is not asserted that a wounded artery will not heal by the efforts of nature alone: Mr. Guthrie knows the contrary. But he is of opinion that such an event should never be contemplated after the recurrence of hemorrhage from it, and that the aid of art is consequently necessary. When an artery is completely divided, it is less likely to continue to bleed, than if it had been only wounded. A variety of opinions have been entertained as to the means employed by nature for suppressing the hemorrhage, as well as those recommended by art. Mr. Guthrie enters at some length into a critical examination of these different doctrines, and he observes,

"In the different theories I have noticed, and especially in that of Dr. Jones, it does not appear that the gentlemen who proposed or maintained them have ever conceived that there was a difference in the means employed by nature, according to the size of the artery injured or divided; that the difference of structure between an artery, such as the carotid or the inguinal, and the tibial or the radial, could cause any deviation from the process they described as taking place, and as they presumed in one invariable manner in all arteries. I shall venture, however, to say that, on the size and variation of structure of the artery, the process employed by nature essentially depends; that it is not the same in large as in small arteries; and that it is not even quite the same in the upper and lower ends of the same artery.

"An artery of moderate dimensions, such as the tibial or brachial, and particularly all below these in size, are in general capable, by their own intrinsic powers, of arresting the passage of the blood through them without any assistance from art, or from the surrounding parts in which they are situated. This overthrows at once the whole theory which relates to the sheath of the vessel and its offices, and in a great measure to the importance derived from the formation of an external coagulum." (P. 222.)

Mr. Guthrie has established the fact both by observation and experiment on man, that arteries in the extremities, of the second order in regard to size, will cease to bleed, through their own efforts, unaided by the assistance of the surrounding parts. He does not mean to contend that the injured vessel is never assisted by them. In many instances, when the artery is divided *in situ,* great assistance is given by the surrounding parts after the retraction of the artery, and from other causes. As a proof that the power and influence of the heart over the circulation through the arteries has been greatly overrated, it is stated that, if the axillary artery be laid bare, before amputating the shoulder, and the surgeon take it between his forefinger and thumb, he will find that almost the slightest possible pressure will be sufficient to stop the current of blood through it. If the artery be fairly divided by the last incision, which separates the arm from the body, without any pressure being made upon it, it will propel its blood with more apparent than real force. A finger placed as lightly as possible against the orifice of the artery, will suppress the hemorrhage. We are even assured by Mr. Guthrie, that if the orifice of the artery happen at the same time to retract and turn a little to one side, so as to be placed in close contact with a solid piece of muscle, the very support thus afforded will prevent its bleeding.

"These are facts, the two first of which I have placed beyond a doubt twenty times in my life. In 1816, I amputated twice at the shoulder-joint in the York Hospital. In neither case did I allow the slightest compression to be made on the subclavian or axillary arteries. On the division of the artery, I first took hold of the divided end between my finger and thumb; then letting it go, I placed the point of my forefinger lightly on the very orifice of the vessel, and stopped the bleeding. In one case, in which I wished a certain loss of blood to take place, I did this twice before nearly a hundred spectators, no one of whom afterwards doubted the fact. So confident am I on this point, that I care not, in this operation, whether a person be ready or not to compress the artery, satisfied that it is always within my own power to prevent any hemorrhage of importance." (P. 227.)

In amputation of the hip-joint, the femoral and profunda arteries bleed furiously, if disregarded, but the slightest compression between the finger and thumb stops both at once. Mr. Guthrie has been taught by nearly all the battles of the peninsular war, to hold all arteries that can be taken between the finger and thumb in great contempt, and he maintains this opinion both by precept and example, publicly and privately, on every occasion. He has lost but one patient from hemorrhage, and in that case a tourniquet was applied. In every point of view, these facts are of the utmost value, and principally because they must tend to relieve the mind of the surgeon from the apprehension of difficulty in arresting hemorrhage.

"It is quite impossible for a man to be a good surgeon, to do his patient justice in great and difficult operations attended by hemorrhage, unless he has this feeling, unless his mind is fully satisfied of the truth of these observations. Whilst his attention is called to more important circumstances, to which the whole of it should be directed, it is perhaps absorbed by the dread of bleeding, by the idle fear that he will not be able to compress the artery, that he may have a dozen vessels bleeding at once, that his patient will die on the table before him. Once fairly in dismay, and the patient is really in danger; but endowed with that confidence, which is only to be acquired through precept and example supported by experience, he surveys this scene with the most perfect calmness, and taking the great artery between the finger and thumb of one hand, he places the points of all the other fingers, of both if necessary, on the next large vessels; or he presses the flaps or sides of the wound together until his other hand is at liberty, in consequence of a ligature having been passed around the principal artery. That it is a scene sufficient to try the presence of mind of any man, I admit; but he is not a surgeon who is not equal to it, who does not delight in the recollection of it when his patient is in safety, and his recovery assured." (P. 228.)

When, however, an artery is only half cut through, and in a situation where the pressure cannot be fairly and equally, although slightly made, the hemorrhage continues in spite of all attempts to suppress it. It is, Mr. Guthrie presumes, from the consideration of such cases, and with the recollection of his own dismay, that the surgeon has been led to suppose that pressure will not stop the current of blood through a large artery. Many cases have occurred to Mr. G. in which the femoral artery has been completely divided; in others, it has only been injured. When the artery has been completely cut across in the *middle or lower* part of the thigh, the patient has either died without assistance, or the hemorrhage has ceased spontaneously. He has not met with an instance in which it was necessary to tie the femoral artery, after it had been divided, and the hemorrhage had ceased for twelve hours, the efforts of nature being sufficient to prevent its return; but he has had several opportunities of examining these arteries when the patient has died of gangrene of the extremity, or of hemorrhage from the lower end of the artery requiring amputation. In the fourth volume of this Journal, several cases which occurred to Mr. Guthrie at the battles of Albuhera and the assault on Badajos, were published, bearing on this point.

"Private J. Barnes, 29th regiment, on the 16th of May, at the battle of Albuhera, received a musket ball in the right thigh, behind and above the knee, inclining downwards and inwards, close to the condyles of the femur, and in the direction of the femoral artery becoming popliteal; which bled violently at the moment, and continued for a few minutes, during which time he conceives he lost two quarts of blood. It then ceased, and he was dressed in the usual slight manner, and remained two days upon the field of battle, until removed to Valverde, nine miles on a bad road, and on men's shoulders, in a blanket converted into a bearer. He was considered as one of the slighter cases, until the gentleman in immediate charge of him requested me to see him, on account of his toes being in a state of mortification, which was considered to point out a more serious injury than was at first supposed.

"On the evening of the 3d of June, eighteen days after the accident, the man was placed on a bullock car, to be removed with the rest of the wounded to Elvas; the mortification of the foot having ceased to increase, and a line of separation having been drawn. Shortly after the cars moved, I was informed that he was bleeding from the wound: it evidently appeared to be the popliteal artery; and, as it flowed slowly, I supposed from the lower divided end. The situation of the wound making the dissection difficult, and the foot being partly lost, I determined on amputation above the knee, which was performed at Olivenca. I caused the amputated limb to be sent after me to Elvas, that it might be examined at my leisure. I carefully traced the course of the wound, and found in it a little coagulated blood, but could not see the mouth of the vessel. A probe passed into the amputated end of the artery was obstructed before it reached the ulcerated surface by near an inch; and on passing it up the lower one, it was stopped exactly in the middle of the track of the ball, by a veil or substance drawn across the mouth of the vessel, and which, on careful examination, showed the point of the probe at one part of the circle, although too small to let it through; and from this part I conceive that the hemorrhage came. The divided ends were one inch apart; each portion was cut out with the ulcerated spot on which they terminated, and opened in their course. The upper, or femoral portion, for near an inch, was filled with a firm coagulum, filling up the contracted mouth of the vessel like the gradual diminution of the neck of a claret bottle; a layer of the same covers the mouth and immediate vicinity, and appears to have a commencing organization. The vein is closed in the same manner. The lower popliteal portion of the artery is very peculiar: the substance diawn across appears to have closed it completely at one time, and to have given way from the rough motion of the car at the point now open, and which is very small even when the sides of the artery are approximated. A very little soft coagulum was behind it; and if the man had not been removed, I think the vessel would have remained secure. This case shows very distinctly the means adopted by nature for the suppression of hemorrhage from either end of a divided artery." (P. 233.)

"Captain St. Pol, of the seventh or Royal Fusiliers, whilst in the ditch at the foot of the breach at Badajos, was wounded in the ham from behind: he fell instantly, and lost, as he thinks, a considerable quantity of blood; he was, on recovering, raised from the ground, and walked a few paces prior to his being carried to his tent, where I saw him in the afternoon of the next day, the 7th. The leg was greatly bent, and turned outwards, and had ceased to bleed before his arrival in camp. A substance could be felt on the inner side of the patella, which, by the sensation communicated to the finger on moving it, appeared to be the ball: it was extracted by a valvular incision, after the manner recommended for the removal of cartilaginous substances found in joints. An artery was accidentally divided, and some dark-coloured blood also issued from the cavity: the ball was lying loose and unconnected; the finger, on being passed in, discovered no splinters of bone; the knee was swelled; and the entrance of the ball, which was a small one, would not admit the finger. His having walked some distance on the leg, and the absence of any splinters between the articulating extremities of the bones, induced Dr. Armstrong, the surgeon, and myself to think that the ball had entered with little injury to the bone; and, after stating to the patient the little hope we had of ultimately saving the limb, independently of the great danger to which he was exposed, compared to the certainty of the operation of amputation at the moment, we consented to his retaining the limb for the present, thinking he would at least get through the inflammatory stage, when the operation could be performed satisfactorily to himself, although under more unfavorable circumstances. Seventeen leeches were applied immediately to the knee, and a cathartic given: the appearance of the leg and foot was natural; no diminution of heat, sensation of pain, or numbness, were felt by the patient, or any thing observed by the surgeons. The next day he was removed into Badajos on a litter, the heats of the tents being insupportable.

"On the morning of the 9th, I saw him early, and the want of circulation in the foot was evident from its having lost its natural colour and warmth; the knee was swelled, but not painful; and it was obvious to me that the artery had been divided by the ball. The marbled appearance and yellow colour soon indicated the loss of the leg above the calf; vesications formed on the foot, already of a green colour.

"On the 12th, the extent of the gangrene was defined on the back of the knee up to the original wound at its lower edge, gradually receding as it advanced to the fore part of the leg, which, for three inches below the knee, was apparently sound; the uneasiness of the knee being moderate, the bites of the leeches and the incised wound looking perfectly healthy, although the latter had not united.

"On the 16th, the separation of the dead from the living parts having taken place from behind, and being well marked and commencing on the fore part, it was agreed in consultation to amputate the limb, which was done about the middle of the thigh. Sixteen vessels were tied, and as little blood as possible was lost. The parts were gently brought together, without much hope of union.

"On the 18th, there being some swelling of the stump, the strips of plaster, already useless, were removed; one remaining as a support to the vessels.

"20th. Half the ligatures came away at once, they having gradually formed into two parcels: the stump was quite open, the bone well covered, and good granulations appearing, with a great discharge of well-formed matter.

"On the 22d, I did not see him, but was informed that the want of strength to carry on the necessary actions became apparent.

"On the 24th, he died.

"On dissecting the amputated limb, the sciatic nerve was found untouched, the ball having passed on the inside; the popliteal vein was also entire, having a small tumor adhering to its under part between it and the artery, the divided end of which I observed closed by a yellowish green firm substance, readily distinguishing it from the surrounding parts. On clearing the whole out from the bone, and making a small circular opening into the tumor, which was elastic and covered with brown fibrous layers, it showed itself to be an aneurismal sac, smooth on the inside, containing florid arterial blood, and some little coagula. The cavity of the sac is not perfectly regular, being nearly divided on one side by a process running into it. The artery, on being carefully slit up to the closed end, appeared to have been injured above the part divided by the ball, and to communicate with the sac by a small fissure or rupture, at the place where the piece of wood is inserted to keep the vessel open. The end of the artery is nearly slit up, so as to show the very little thickness of the closing substance, and the great original contraction of the diameter of the vessel. There was no internal coagulum, neither was there any laid over the external part of the artery; between it and the bone there was a coagulum lying, of the size of a small phial-cork. Displeased with myself for having listened to the petition of hope, which I knew, even under more favorable circumstances, would be futile, I felt some satisfaction on viewing the diseased parts, which showed that process of nature completed which in the former case had only been begun, although a longer period of time had elapsed." (P. 237.)

It was supposed that the aneurismal sac existed prior to, and was independent of, the gunshot wound, as a sac of that nature could not be formed in ten days. Dr. Armstrong recollects that Captain St. Pol complained once or twice of uneasiness in the knee, previous to his being wounded, but in so casual a manner as not to have caused further inquiry. The other end of the artery could not be found, from the state of the parts.

Although mortification does not occur in all cases after injury of the femoral artery, it is always to be dreaded. Mr. Guthrie mentions it as a curious and interesting fact, that the *lower* end of a divided artery is more prone to secondary hemorrhage than the upper; so much so, that when bleeding occurs after having been arrested for a period of four hours, it takes place, in all probability, from the lower end.

"This may always be known from the darker colour of the blood, and from its flowing out in a continuous stream, in the same manner as water rises from a spring, and not with any arterial impulse. The surgeon has no right to believe that the blood comes from the upper extremity of the artery, unless it is of a florid, scarlet, arterial colour, when it will usually rush out with force, if not with the undisguised arterial impetus. This cannot be an accidental circumstance; it has happened much too often to be attributed to such a cause, and it has appeared to me to arise from a difference in the process adopted by nature in one end of the artery to that in the other. It is a point to which I paid particular attention during the whole war, and which I have since made the subject of experiment. I cannot be mistaken as to the fact, although I may err in the explanation." (P. 248.)

Mr. Guthrie believes that the retraction or contraction is not so complete in the lower as in the upper end of the vessel, and he is certain they are not so permanent. He has been led to suspect also, from observation and experiment, that the internal coagulum of blood is not so perfectly formed in the lower end of a divided artery.

"The same kind of yellowish green matter marks and covers the situation of the lower extremity of the artery, as it does the upper: it is, however, thinner where it immediately covers the end of the artery, which in none of these cases was contracted in the conical manner I have described as occurring in the upper extremity of the vessel. On the introduction of a probe into the artery with the greatest gentleness from below, it made its appearance at a point on the yellow space, raising a thin portion of it as it protruded. On laying open the artery, the orifice seemed to have been once closed by this layer of fibrine or lymph, but without a degree of contraction corresponding to that observable in the upper end of the same artery; the layer still forming an obstacle sufficient to cover and close three fourths of the orifice, the blood having flowed through the remaining fourth.

"These appearances seem to indicate a different process in nature to that adopted for the closure of the upper end of the vessel; and the frequency of their occurrence, to demonstrate that the process is a natural one. I have been led to conclude that the retraction and contraction of the lower end of a divided artery is neither so perfect nor so permanent as at the upper end; and that the internal coagulum is in many instances altogether wanting, or very defective in its formation; giving rise to a very different result from that which is observable in the upper divided end of the same vessel." (P. 250.)

When an artery is merely cut or torn, but not quite divided, it is in the same state, with regard to hemorrhage, as if it had given way by ulceration. It can neither retract nor contract, and will continue to bleed unless pressure be accurately applied and maintained, until it destroys the patient. The practice to be pursued is to divide the vessel if it be a small one, as the temporal artery; when it will retract and contract, and bleeding soon ceases. If an artery of larger dimensions be wounded, a ligature should be applied above and below the wound, and the vessel may be divided or not, at the pleasure of the surgeon.

*On the Method of performing Operations on Wounded Arteries.* Mr. Guthrie has, in his previous works, clearly shown the impropriety of applying the theory of the operation for aneurism to wounded arteries. It has been concluded by many surgeons that the same methods of operating, the same directions, and the same mode of proceeding, would suit the injury as well as the disease. The brilliant theory of the cure of aneurism by the Hunterian method, and the more brilliant operations which followed it, seemed to have effaced the recollection of the ancient methods of proceeding in regard to wounded arteries; and it was not until repeated failures had convinced every one that the theory which would apply in cases of aneurism would not apply to wounded arteries, that it was abandoned, and the old method was resorted to of tying both ends of the wounded vessel. For the restoration of the proper mode of proceeding in such cases, we are in a great measure indebted to Mr. Guthrie. But, although the principal was abandoned, the *modus operandi* has been retained, as if the operation were for aneurism, and not for a wounded artery.

"In an operation for aneurism, the directions are all laid down with precision: a just proportion of the common integument is to be divided, and no more; then so much fascia of various kinds; then such a muscle is to be turned, dissected inwards or outwards as the case may be, or detached, but on no account is it to be divided; and the operation is to be finished by applying the ligature in the particular place which has been exposed by these proceedings. Nothing can be more proper in a case of aneurism; but the wound of an artery is an accidental occurrence, it does not happen always in the particular place which will suit these directions: nevertheless, they are to be adapted to it in the best way that can be thought of, and the young surgeon is left to his discretion. The principal error in this method of proceeding, as adapted to wounded arteries, arises from a strange and unaccountable fear of cutting muscular fibres, which seems to pervade the minds of all the surgeons of the present day who have treated on these subjects. A dread so truly ludicrous, that it appears incomprehensible how it can possibly have continued until the present time. I shall suppose that a gentleman of rank and importance in the country is struck on the back part of the leg by a very sharp cutting instrument, which divides the muscular fibres of the gastrocnemius and soleus muscles in a longitudinal direction, cuts across the tendon of the plantaris, but does not injure either the fibular or posterior tibial artery, or its nerve. I shall further suppose that any one of the best anatomists and surgeons in London is sent for, and his opinion desired as to the result of the case: I venture to assert that he would say, his patient was a most fortunate man to have suffered so little injury from such a wound; for if the posterior tibial artery or nerve had been divided, he might have bled to death, or have been lamed for life; but that, under existing circumstances, he hoped, by position, quietude, compress, bandage, &c., that is, by a due application of the usual means, to be able to reunite the wounded parts; so that, in all probability, unless some untoward circumstances took place, the gentleman would soon recover, and without any permanent defect or lameness. No man of knowledge could say otherwise. Let us now suppose that the same gentleman, having been cured of the accident according to the opinion of his surgeon, has the misfortune to be wounded by a pistol ball in a duel whilst standing sideways towards his opponent; so that the ball passes across the calf of the leg, close behind, but not injuring either bone, although dividing the fibular or posterior tibial artery, which bleeds in such a manner as to require the ligature: we will also suppose the same surgeon in attendance: what operation would he do, and how would he set about it? I will take the liberty of suggesting that, in a case of this kind, blood would flow from both wounds, and that a surgeon acquainted with his profession would first endeavour to ascertain which vessel was wounded. This he could only do by enlarging one or both openings, so as to enable him to introduce his forefinger into each, the point of which might, from its sensibility, detect the wound through the impetus of the flow of blood, if it happened to be placed on the cut part of the artery. In this manner he might possibly be enabled to judge with accuracy which of the vessels was wounded, although it might very readily happen that both were injured and both were bleeding. The minor disaster will answer our purpose, and one vessel only shall be wounded. Shall he, with the Baron Dupuytren, tie the femoral artery as for aneurism, or shall he, on the other hand, cut down and tie both ends of the artery?" (P. 255.)

If the latter operation be determined upon, the mode in which it will be performed is objectionable, if those rules are complied with which are laid down in the best surgical works. A difficult, tedious, and dangerous operation is recommended, Mr. Guthrie contends, solely because it is not usual to make a longitudinal incision in the muscles of the calf of the leg; an incision which, if made by accident, would not be deemed dangerous or likely to lead to any subsequent detriment.

"An incision is to be made six or seven inches in length, by successive and rapid incisions, through the integuments and muscles of the calf of the leg down to the fascia. The centre of the incision is to be on a line with the shot holes, or if they are diagonal to each other, between them; and it may be either directly in the middle of the calf, or a little to the side of, or directly over the artery supposed to be wounded: it is not material which. The smoothness of the fascia points it out, and the loose cellular membrane connecting the divided muscles to it, allows of the edges of the long incision being easily separated, and to such a distance as to admit of the exposure of the great nerve, the arteries, and veins, in as distinct a manner as any other arteries, veins, and nerves can be exposed in the human body. The tourniquet is now to be unscrewed, and the bleeding, if the wound did not bleed before, leads to the spot where the artery is injured. The knife may be applied perpendicularly to the fascia, and the artery laid bare for three or four inches in extent, by as common a piece of dissection as any ever practised, and nothing can interrupt the application of the ligature. The nerve and the fascia cease to be surgical bugbears, and the operation is as simple as any in surgery. No surgeon or anatomist will dispute this statement: he may, however say that the muscles have been divided, and that surgeons have not been in the habit of cutting through them by a fair incision in their length; that they have hitherto only done it by insinuating a director under the head of the soleus, and separating it from its attachment to the bone; as if the separation of a muscle from its bony attachment was not much more likely to lead to weakness and defect in the action of that muscle, than a mere interstitial incision in a longitudinal direction." (P. 260.)

Mr. Guthrie goes even a step further, and shows that a muscle may be cut transversely to its course or fibres, whenever it may be desirable to do so, to place a ligature on a wounded artery beneath. He proves also that little or no inconvenience results from such practice.

"Lieutenant-Colonel Wildman, now of the Carabineers, was wounded at the battle of Albuhera, by a sabre cut directly across the middle of the deltoid muscle, so as to divide it completely down to the bone of the arm. He was taken prisoner at the same moment, and the French surgeons dressed his wound by filling it with charpie, and then bound the arm down to the side. He made his escape in the night, and was brought to me the second day after the accident. The first thing I did was to remove the dressings, raise the arm to a right angle with the body, by which alteration the edges of the wound might be brought into contact, and then try, by a continuation of the position, and by compress and bandage, to keep the parts together, so that as little deficiency of substance might appear as possible. The wound did not unite under this treatment by the first intention, but it readily did so by granulation, and his recovery is so complete that at this moment he is unconscious of any defect in strength or motion whatsoever.

"At the battle of Salamanca, a French soldier received a sabre cut, vertically and directly across the fibres of the pectoralis muscle, at the inner side of the vessels; the lower and fore edge of the muscle was completely divided. The man had other wounds, but this one was never considered but as a simple one, and by proper compress and bandage, it united so as to leave merely a little weakness of no consequence." (P. 262.)

Another point is discussed; namely, the manner of securing the ulnar artery, when wounded a little below its origin, when it is covered by the pronator teres, and the superficial flexors of the forearm. By high authority, we have been told that, "in the superior third of the forearm, the great depth at which this artery lies from the surface, and the number of muscles which cover it, render it impracticable to pass a ligature around it." Mr. Guthrie asks, why is it impracticable? and he answers, that the artery cannot be laid bare unless the muscles above mentioned are divided, and that there is no reason why this should not be done. Cases of this sort are referred to, in which the lives of patients have been lost from the fear or unwillingness of the surgeon to divide a few muscular fibres.

"It is necessary now to ask what would be the consequence of this division? The utmost consequence which could ensue would be weakness of the arm in the performance of certain motions: but I have no hesitation in affirming that no such consequence would ensue. I have seen the parts divided, nay, I have divided them myself, and the patient has recovered without any sensible defect. The following case is a strongly marked one on this subject: it was from it that I first drew this confirmation of my ideas on wounded arteries.

"The corporal of pioneers of the twenty-ninth regiment, was wounded at the battle of Rolica by a musket-ball, which passed through the anterior and upper part of the forearm, fracturing the ulna. A few days after the injury, an artery sloughed, and bled profusely. The surgeon in charge immediately tied the brachial artery above the wound, and in the night the hemorrhage recurred, and the man nearly bled to death. The arm was now amputated, and the ulnar artery was found in an open and sloughing state.

"This man's arm would have been saved, if an incision had been made down to the artery, taking the shot hole as a centre, and then, when the vessel was laid bare, placing a ligature on each of its divided ends.

"At the battle of Vimiera, which followed a few days after, a soldier received a similar wound, save that the artery bled forthwith, and could only be stopped by the continued application of a tourniquet. I cut down on the artery, through all the intervening parts, carefully avoiding the median nerve, and found the artery more than half divided; a ligature above and below the wound suppressed the hemorrhage, and it did not afterwards return." (P. 272.)

Mr. Guthrie animadverts keenly, but very fairly, on the practice recommended by M. Dupuytren, in a memoir he published in the Repertoire Generale, &c. tome v. 1828: "Sur les Anévrysmes qui compliquent les Fractures," &c. M. Dupuytren applies the theory of Hunter's operation for aneurism to the treatment of a wounded artery; but, as upon this point we have already stated the opinions of Mr. Guthrie, it is not necessary formally to show that he cannot agree with the practice recommended in the memoir.

Mr. Guthrie next offers some interesting practical observations on the best mode of proceeding in those cases in which hemorrhage takes place from an artery that cannot be tied at the part where it is wounded.

A few pages are devoted to aneurism by anastomosis; and the volume concludes with directions for securing various arteries.

The reputation of Mr. Guthrie as an acute observer, his extent of experience, and his indefatigable zeal in the improvement of his profession, are so well known, that every work that proceeds from his pen is sure to meet with attention. We need only say, that the present volume abounds with interesting information to the surgeon.

